# Flexible and static wrist units in upper limb prosthesis users: functionality scores, user satisfaction and compensatory movements

**DOI:** 10.1186/s12984-016-0130-0

**Published:** 2016-03-15

**Authors:** M. Deijs, R. M. Bongers, N. D. M. Ringeling - van Leusen, C. K. van der Sluis

**Affiliations:** Department of Rehabilitation Medicine, University Medical Center Groningen, University of Groningen, P.O. Box 30.001 (CB41), 9700 RB Groningen, The Netherlands; Center for Human Movement Sciences, University Medical Center Groningen, University of Groningen, Antonius Deusinglaan 1, 9713 AV Groningen, The Netherlands; Revant Rehabilitation Center, Brabantlaan 1, 4817 JW Breda, The Netherlands

**Keywords:** Prosthetic limbs, Wrist, Functionality, User satisfaction, Compensatory movements

## Abstract

**Background:**

The current study examines the relevance of prosthetic wrist movement to facilitate activities of daily living or to prevent overuse complaints. Prosthesis hands with wrist flexion/extension capabilities are commercially available, but research on the users’ experiences with flexible wrists is limited.

**Methods:**

In this study, eight transradial amputees using a myoelectric prosthesis tested two prosthesis wrists with flexion/extension capabilities, the Flex-wrist (Otto Bock) and Multi-flex wrist (Motion Control), in their flexible and static conditions. Differences between the wrists were assessed on the levels of functionality, user satisfaction and compensatory movements after two weeks use.

**Results:**

No significant differences between flexible and static wrist conditions were found on activity performance tests and standardized questionnaires on satisfaction. Inter-individual variation was remarkably large. Participants’ satisfaction tended to be in favour of flexible wrists. All participants but one indicated that they would choose a prosthesis hand with wrist flexion/extension capabilities if allowed a new prosthesis. Shoulder joint angles, reflecting compensatory movements, showed no clear differences between wrist conditions.

**Conclusions:**

Overall, positive effects of flexible wrists are hard to objectify. Users seem to be more satisfied with flexible wrists. A person’s needs, work and prosthesis skills should be taken into account when prescribing a prosthesis wrist.

**Trial registration:**

Nederlands Trial Register NTR3984.

## Background

Wrist movement is an important requirement in upper limb prosthesis design for successfully employing the prosthesis in activities of daily living (ADL) [1–3]. However, most upper limb prostheses are currently equipped with wrists that can only rotate, either passively by using the non-amputated hand, or actively through a myoelectric signal. Although several prosthesis wrists with multiple passive motion capabilities such as flexion/extension and radial/ulnar deviation are commercially available (e.g. Flex-wrist, Otto Bock®; Multi-flex wrist, Motion Control®; Michelangelo Hand, Otto Bock®; FW Flexion Friction Wrist, Hosmer®), their contribution to functionality or user satisfaction has hardly been investigated.

When rotation is the only possible movement in the wrist, the position of the hand in space is mainly determined by the elbow, shoulder and trunk. Several studies reported changed movement patterns in proximally located body segments of users of prosthesis wrists with only a rotation function [4–11]. These compensatory movements may lead to musculoskeletal complaints [10, 12] or overuse injuries [13].

From the scarce literature on flexible wrist units in prostheses, it is suggested that the use of flexible wrists may lead to a decrease in compensatory movements and an increase in functionality. From a study on motion patterns typically performed in patients’ daily living [5], it was concluded that compensatory movements, especially at the shoulder on the prosthetic side, were reduced by wrist flexion. Also, patients perceived that increased flexibility of allowed wrist motion optimized their motion pattern. From a preliminary field trial on patients using the Multiflex-wrist (Motion Control), it was reported that patients experienced an improvement of the overall comfort of the prosthesis while performing activities over the course of a day, and that the ease of performing tasks improved [14]. Another study of an able-bodied subject using a flexible wrist with a prosthesis simulator supports the expectation that prosthesis users may handle faster and easier with their prosthesis [4]. Some tasks were performed quicker and with less difficulty as a result of wrist flexion, reflected in higher functional scores on the Southampton Hand Assessment Procedure (SHAP).

However, these studies included only a few prosthesis users [5, 14] or an able-bodied person [4], and focused each on only one outcome measure (i.e. compensatory movements, user satisfaction or functionality). The aim of the current study was to assess differences between flexible and static prosthesis wrists on functionality, user satisfaction and compensatory shoulder movements in prosthesis users with a transradial upper limb deficiency.

## Methods

### Participants

Prosthesis users were selected from databases of the orthopedic workshop OIM Orthopedie, the Department of Rehabilitation Medicine from the University Medical Center Groningen and Revant Rehabilitation Center Breda, all in the Netherlands. Prosthesis users were eligible if they (1) had an acquired amputation or a congenital deficiency at transradial level, (2) had at least one year experience with a myo-electrical prosthesis with a wrist unit that can only rotate; (3) wore their prosthesis for at least four hours a day; (4) had a prosthesis hand with one degree of freedom (opening and closing the hand); (5) did not have experience with a flexible wrist unit. Exclusion criteria were co morbidities that could influence the test performance (e.g., neurological disorders or rheumatic diseases that can influence arm function) and insufficient command of the Dutch language to fill in questionnaires.

To enable assessment of compensatory shoulder movements, able-bodied control subjects were recruited by advertisements and were included when they matched with the prosthesis users for hand dominance (prosthesis user’s dominant hand was considered as the non-affected hand), gender, age, height and weight (±10 years, ± 10 cm, ± 10 kg), and were free of musculoskeletal complaints.

Participants and control subjects provided their written informed consent and were rewarded with a gift voucher when completing the experiment. The study was approved by the Medical Ethical Committee of the University Medical Center Groningen (NL44256.042.13).

### Study design

Eight prosthesis users tested two different wrist units in a cross-over design (Table 1). The wrists could only be used with the corresponding hand: Flex-wrist with transcarpal hand (Otto Bock®) and Multiflex-wrist with 7 ¾ ProPlushand (Motion Control®). The Flex-wrist (Otto Bock®) could be secured in a neutral position and in 20 and 40 degrees of flexion as well as extension. The Multiflex-wrist (Motion Control®) could be secured in a neutral position and in 30 degrees of flexion and extension. Furthermore, the latter wrist could move freely in flexion and extension direction. In all positions, locked or unlocked, free radial and ulnar deviation was present in the Multiflex wrist. The wrist unit was spring loaded to the center of travel, so it could flex in multiple directions as needed to adjust to the loads placed on it, and thus adapted to changes in direction and pressure. Attaching and detaching of both wrists was performed with the non-affected hand by pressing a button on the wrist.

**Table 1:** Schematic overview of the study design

Experiment															Follow-up
P		2 weeks			2 weeks			2 weeks			2 weeks			1 month	
1	*T0*	FW	s	*T1*	FW	f	*T2*	MW	s	*T3*	MW	f	*T4*		*T5*
2	*T0*	FW	s	*T1*	FW	f	*T2*	MW	f	*T3*	MW	s	*T4*		*T5*
3	*T0*	FW	f	*T1*	FW	s	*T2*	MW	s	*T3*	MW	f	*T4*		*T5*
4	*T0*	FW	f	*T1*	FW	s	*T2*	MW	f	*T3*	MW	s	*T4*		*T5*
5	*T0*	MW	s	*T1*	MW	f	*T2*	FW	s	*T3*	FW	f	*T4*		*T5*
6	*T0*	MW	s	*T1*	MW	f	*T2*	FW	f	*T3*	FW	s	*T4*		*T5*
7	*T0*	MW	f	*T1*	MW	s	*T2*	FW	s	*T3*	FW	f	*T4*		*T5*
8	*T0*	MW	f	*T1*	MW	s	*T2*	FW	f	*T3*	FW	s	*T4*		*T5*

*Abbreviations*: *FW* Flex-wrist, *MW* Multi-flex wrist, *s* static condition, *f* flexible condition, *P* participant, *T0-T5* measurement moments

Note: All participants used their own prosthesis wrist and hand again in the month prior to T5

Wrists and hands were attached to the participant’s own prosthesis socket using a standard quick-disconnect system. Participants received both wrists and the corresponding hands on a loan basis, and used them as much as possible in their home environment, during work or during leisure activities. All wrist units were used in their flexible and static conditions for a two week period per condition (Table 1). The order of the four conditions (Flex-wrist, static; Flex-wrist, flexible; Multi-flex wrist, static; Multi-flex wrist, flexible) was balanced over participants by randomly providing them a subject number generated in Matlab, Mathworks, R2007a. In the static condition, the wrists were secured in their neutral positions. In the flexible condition, participants were encouraged to explore all possible positions of the wrists, locked or unlocked. Participants received oral and written instructions to operate both wrists. To ensure and encourage sufficient use of the prosthesis and thus sufficient exploring of using the new prosthesis wrist, participants were asked for the amount of time they used their prosthesis and corresponding wrist at every measurement, and in a phone call halfway each two week period in which a particular wrist was used.

### Measurements

At the start of the experiment, a baseline measurement T0 was conducted using the participants’ own prosthesis wrist and hand. After using a prosthesis wrist in a certain condition for a two week period, follow-up measurements T1-4 took place (Tables 1 and 2). A final measurement was performed about one month after completion of the experiment (T5). All participants used their own prosthesis wrist and hand again in the month prior to T5.

**Table 2:** Overview of measurement instruments for participants (prosthesis users) and control subjects

Level	Instrument	Participants	Control subjects
General information	General questionnaire	T0	T0
Functionality	SHAP	T0-T4	
	Box and Block test	T0-T4	
	UEFS 2.0 (OPUS)	T0-T4	
Satisfaction	D-Quest (assistive device subscale)	T0-T4	
	TAPES	T0-T4	
	3 additional items to TAPES	T0-T4	
	Open ended questions on (dis)advantages	T0-T4	
	Semi-structured interview	T5	
Shoulder movements	Check of shoulder range of motion	T0	T0
	Shoulder movement measurement in ADL tasks	T0-T4	T0

At T0, prosthesis users handed in a questionnaire they had filled out at home. Questions concerned personal information like gender, age, side of amputation or deficiency, cause of short arm, time since amputation, experience with myo-electric prostheses, occupation and co morbidities.

Measurements T0-T4 consisted of questionnaires and open-ended questions to measure satisfaction and functionality with the different wrist units. To assess functionality further, SHAP and Box and Block tests were applied. Finally, shoulder movements were measured during execution of 6 ADL tasks. To provide for reference values for the movements with the prostheses, a group of able-bodied control subjects performed the same ADL tasks as the amputee participants.

In all these measurements the same prosthesis wrist and hand were used as that the participants had used in the previous two weeks (Table 2). The tests were performed in a random order that differed on each measurement and for each participant. During testing, participants were free to adjust wrist rotation and, if the flexible wrist condition was tested, they were free to adjust the flexion and extension position of the wrist according to their preferences. Measurement T5 consisted of a semi-structured interview.

#### Functionality

At T0-4 the following measurements regarding functionality were performed:

- SHAP. The Southampton Hand Assessment Procedure consists of 26 tasks: 12 abstract tasks and 14 ADL. The time needed to complete a task is recorded. The Index of Function score (0–100) is generated, in which a score of 98 represents a normal hand function [15, 16].
- Box and Block (B&B) test. The B&B is performed with both the unaffected hand and the prosthesis hand. The participant has to move as many as possible cubes from one box to the other within one minute. The amount of cubes moved within a minute indicates how competent the participant is with the prosthesis in grabbing, holding, moving and letting go of the cubes [17].
- UEFS 2.0 (Upper Extremity Functional Status), part of the OPUS (Orthotics and Prosthetics Users’ Survey) [18]: a 19-item questionnaire (score 0–57) which assesses upper extremity function by scoring how easily ADL tasks are performed. Higher scores reflect better functionality.

#### Satisfaction

At T0-4, the following measurements regarding satisfaction were performed:

- D-QUEST (Dutch version of the Quebec User Evaluation of Satisfaction with assistive technology), subscale ‘assistive device’ [19]. This questionnaire measures user satisfaction with the assistive device in eight items (i.e. dimensions, weight, ease in adjusting, safe and secure, durability, ease of use, comfort, effectiveness) on a five-point scale. A higher score on the subscale (scores range from 5–40) represents a higher satisfaction. The complete D-QUEST was found to be reliable and valid to assess user satisfaction of various adaptive devices [20].
- TAPES (Trinity Amputation and Prosthesis Experience Scale), prosthesis-satisfaction subscale [21]. This questionnaire measures satisfaction with the prosthesis in general in ten items on a five-point scale.
- Three additional items, namely ‘moving naturally’, ‘wearing and tearing of clothes’, and ‘ease when performing tasks’ were administered using the same five-point scale as the TAPES.
- Open-ended questions. Participants were asked to write down the two most important advantages and the two most important disadvantages of the wrist they used in the past two week period.

At T5, a semi-structured phone interview was conducted, to reflect on the most important advantages and disadvantages of both types of prosthesis wrists and which wrist unit was preferred by the participants.

#### Compensatory movements

At T0, shoulder joint mobility of amputee participants and control subjects was checked by asking for maximum active abduction and applying maximum passive abduction and external rotation.

During the execution of six ADL tasks (see Appendix), shoulder angles were measured using Inertial & Magnetic Measurement System (IMMS) [22]. Therefore, MTw™ sensors (MTw™ sensors, Xsens Technologies, Netherlands; manufacturer specifications: angular resolution = 0.05 deg, static accuracy (roll/pitch) <0.5 deg, static accuracy (heading) = 1 deg, dynamic accuracy = 2 deg RMS) were placed on the thorax-sternum, latero-distally on the humerus of both arms, and on both scapulae. The sensors were attached using tape or Velcro strips. Also, joint movements were videotaped using a hand camera.

The ADL tasks were performed in a randomized order. Calibration of the MTw™ sensors took place by asking participants to stand or sit in a pre-defined posture, which was also the starting posture for a specific task. In tasks performed while standing, the starting posture was defined as ‘standing upright and holding the arms in a relaxed way alongside the body’. In tasks performed while sitting, the starting posture was defined as ‘sitting upright at a distance from the table with the elbows bended in 90 degrees and the wrists positioned at the edge of the table in a neutral position’. Participants were allowed to practice the task once. Subsequently, each ADL task was repeated four times. Participants were instructed to return to their starting posture after each trial. Where prosthesis users performed tasks with their prosthesis hand, control subjects performed the task with their non-dominant hand.

### Data analysis

#### Functionality

Visual inspections of normal Q-Q plots and results from the Kolmogorov-Smirnov test of normality indicated that scores on SHAP and Box and Block test did not significantly differ from a normal distribution in all conditions. Therefore, repeated measures ANCOVA with factors type (Flex-wrist and Multi-flex wrist) and condition (flexible and static) was used to analyse differences between the wrist conditions on the scores of both SHAP and Box and Block test (note that for Box and Block test, only the scores obtained with the prosthesis hand were included in the analysis). The condition ‘own prosthesis’ was included as a covariate.

A Friedman test in which the four wrist conditions were included, was used to analyse differences between the wrist conditions on UEFS 2.0 (OPUS).

#### Satisfaction

Friedman tests were used to analyse differences between the four wrist conditions on D-QUEST, TAPES, and our three additional items to TAPES.

Sound recordings of the semi-structured interviews (T5) were transcribed verbatim. Three authors (MD, NR, and CS) highlighted what they perceived as important and remarkable quotes in the interviews, focusing on advantages and disadvantages of the prosthesis wrists. One author (MD) extracted the advantages and disadvantages of the prosthesis wrists from these highlighted responses together with the written responses on the open-ended questions (T0-T4). Also, it was documented which wrist participants would choose if they could have a new prosthesis, and why. Eventually, the table was checked for consensus by the other two authors (NR and CS).

#### Compensatory movements

By multiplying the inverse rotation matrices of the MTw™ sensors of the trunk by the rotation matrices of the upper arm sensors, the rotation matrices of the shoulder angles were computed at each timeframe. Because we often found gimbal lock when computing Euler angles following [23], we computed attitude vectors [24]. Euler angles, which are conventionally reported in rehabilitation and movement science, decompose a rotation from one pose to another into three rotations, each around an axis of an orthogonal local reference frame. Although usually represented in the literature with three angles, a rotation in a joint of the arm such as the shoulder joint is actually a rotation around one axis through that joint. The attitude vector represents the orientation of that axis and the length of the attitude vector reflects the amount of rotation with respect to the axis. We computed the length of the attitude vector at each timeframe, over the course of the movement. The maximum values of the attitude vectors within each trial were averaged over the repetitions in each task for each participant, reflecting the maximum angles of rotation in the shoulder (i.e., the rotation angle). If this angle of rotation was larger, the degree of movement in the shoulder was larger. We compared these angles of rotation for the different conditions to assess whether more compensatory movements were performed. Descriptive statistics were presented for these maximum angles of rotation in the shoulder for all tasks in all conditions of the prosthesis users and for the control subjects.

All statistical analyses were conducted using IBM SPSS Statistics 20, using a significance level of α = 0.05.

## Results

After retrievals from the available databases, 32 prosthesis users in total were eligible for the study. Initially, 25 prosthesis users were sent an invitation letter. The other prosthesis users (*n* = 7) were placed on a reserves list due to e.g. large travel distance. After this first round, 11 prosthesis users returned their informed consent form. Eventually, three prosthesis users of this group were not satisfying the selection criteria and one prosthesis user withdrew due to personal circumstances after initial consent. After sending (reminder) letters to non-responders and reserves, two prosthesis users responded of which the first one was selected as the final participant. Eventually the targeted eight amputee participants (six men, two women; mean 50, s.d. 14 years) were included (Table 3).

**Table 3:** Participant characteristics

Gender	Age (years)	Cause of upper limb defect	Side of upper limb defect	Original preferred hand	Time after amputation (years)	Experience with myoelectric prosthesis (years)	Own prosthesis hand (manufacturer)	Type of work	Co morbidities
F	24	ULRD	left	N.A.	N.A.	20	Otto Bock	office	-
M	43	ULA	left	right	10	10	Otto Bock	office/hand work	respiratory disease
M	44	ULRD	right	N.A.	N.A.	22	Otto Bock	office	cardiovascular disease
M	49	ULA	right	left	21	>7^a^	Otto Bock	hand work	musculoskeletal complaints
M	54	ULA	left	both	12	10	Otto Bock	unemployed	-
M	55	ULA	right	right	22	22	Otto Bock	hand work	-
F	62	ULRD	left	N.A.	N.A.	5	Motion Control	unemployed	musculoskeletal complaints
M	71	ULA	right	right	50	30	Otto Bock	retired	cardiovascular disease

*Abbreviations*: *F* female, *M* male, *N.A*. not applicable, *ULRD* upper limb reduction deficiency, *ULA* upper limb amputation

^a^ experience unknown, but more than 7 years

The wearing time of the prostheses and corresponding wrists in the four conditions was evaluated as the usual amount of time by 5 to 7 participants. One to 3 participants revealed that they wore the prosthesis less than average due to the following reasons: holidays, socket irritation, defect of prosthesis hand, fear for damaging the hand during work, or illness.

### Functionality

No significant differences between static and flexible wrist conditions were found on SHAP (F(1,6) = 2.74, *p* = 0.15), Box and Block test (F(1,6) = 0.47, *p* = 0.52) and UEFS 2.0 (*χ*^2^(3) = 1.33, *p* = 0.72) (Table 4). Some, but not all participants obtained a higher score on the functionality tests when using the prosthesis wrist in a flexible compared to static condition. However, the Coefficient of Variation (CoV) showed that the inter-individual variation was considerable: as low as 15 % and as high as 50 % (Table 4).

**Table 4:** Mean scores on SHAP, Box and Block, UEFS 2.0, D-QUEST and TAPES for all wrist conditions

	Own prosthesis	Flex-wrist (static)	Flex-wrist (flexible)	Multi-flex wrist (static)	Multi-flex wrist (flexible)
Functionality					
SHAP	55 ± 17(31 %)	58 ± 11(18 %)	56 ± 17(30 %)	51 ± 15(30 %)	53 ± 17(33 %)
Box and Block	14 ± 7(49 %)	16 ± 8(47 %)	18 ± 6(34 %)	16 ± 8(50 %)	17 ± 7(44 %)
UEFS 2.0	48 ± 7(15 %)	45 ± 8(18 %)	47 ± 8(17 %)	46 ± 7(16 %)	47 ± 8(16 %)
Satisfaction					
D-QUEST	30 ± 5(15 %)	28 ± 5(20 %)	28 ± 4(15 %)	28 ± 6(21 %)	29 ± 7(24 %)
TAPES	37 ± 7(18 %)	36 ± 7(19 %)	35 ± 6(18 %)	36 ± 5(15 %)	37 ± 7(20 %)

Note: Scores are presented as mean ± s.d. (standard deviation) with coefficient of variation in parentheses. Higher scores on SHAP, Box and Block and UEFS 2.0 reflect better functionality. Higher scores on D-QUEST and TAPES reflect higher satisfaction

### Satisfaction

No significant differences between wrist conditions were found on the standardized questionnaires regarding satisfaction, namely D-QUEST (*χ*^2^(3) = 1.36, *p* = 0.72) and TAPES (*χ*^2^(3) = 0.94, *p* = 0.82), and on our three additional items to TAPES (*χ*^2^(3) = 2.58, *p* = 0.46 for moving naturally; *χ*^2^(3) = 3.75, *p* = 0.29 for wearing and tearing of clothes; *χ*^2^(3) = 1.86, *p* = 0.60 for ease when performing tasks) (Table 4). A detailed overview of participants’ satisfaction with the devices and their different conditions is shown in Table 5, in which the mean scores on individual items of the D-QUEST are shown. A trend of improved scores for flexible compared to static conditions can be seen on the items ‘ease in adjusting’ (in both Flex-wrist and Multi-flex wrist) and ‘ease of use’ (in the Multi-flex wrist).

**Table 5:** Mean scores on the items of the D-QUEST for all wrist conditions

	Own prosthesis	Flex-wrist (static)	Flex-wrist (flexible)	Multi-flex wrist (static)	Multi-flex wrist (flexible)
Dimensions	4.1 ± 0.4	3.4 ± 1.2	3.5 ± 1.2	3.6 ± 1.1	4.0 ± 0.5
Weight	3.8 ± 1.0	3.9 ± 0.8	3.9 ± 0.8	3.4 ± 1.3	3.5 ± 1.6
Ease in adjusting	3.1 ± 1.1	2.9 ± 1.1	3.6 ± 0.7	2.9 ± 1.1	3.5 ± 0.9
Safe and secure	4.0 ± 0.8	3.6 ± 0.9	3.3 ± 1.2	3.5 ± 0.8	3.5 ± 1.3
Durability	3.6 ± 0.9	3.5 ± 0.9	3.5 ± 0.9	3.5 ± 0.8	3.4 ± 0.7
Ease of use	4.1 ± 0.6	3.8 ± 1.0	3.6 ± 0.7	3.5 ± 1.1	4.0 ± 0.9
Comfort	3.6 ± 0.5	3.2 ± 1.3	3.4 ± 0.7	3.5 ± 0.9	3.5 ± 1.2
Effectiveness	4.0 ± 0.9	3.6 ± 0.7	3.6 ± 0.7	3.8 ± 0.9	3.5 ± 1.1

Note: Scores are presented as mean ± s.d. (standard deviation). Higher scores reflect higher satisfaction

In general, participants’ satisfaction, derived from the written responses on the open-ended questions and from the interviews, tended to be in favour of flexible wrists. In the interview, all participants but one indicated that they would choose a prosthesis hand with wrist flexion/extension capabilities if they could get a new prosthesis (Table 6). The preference for a Flex-wrist or Multi-flex wrist mainly depended on the personal preference for either stability (Flex-wrist) or flexibility (Multi-flex wrist). In general, stability, robustness, trust, support, and no fear for unexpected movements are mentioned as advantages of a completely static wrist. Participants who experienced these aspects as advantages of static wrists, named a lack of stability, fear for unexpected movements and missing ones hold as disadvantages of flexible wrists. At the same time, they acknowledged that it may require habituation. Participants who experienced the stiffness, restrictions in movements and awkward postures with a static wrist as significant disadvantages, stated that using flexible wrists resulted in a more natural movement pattern, less awkward postures, less burden in the shoulder and increased dexterity, and thus may be a solution for their problems with static wrists. Employment was also a contributing factor for wrist preferences: employed participants underlined the importance of suitability of the prosthesis wrist in their activities at work. In addition, participants who were unemployed or retired indicated that they might have benefitted more from the flexible wrist if they would still have been employed in a job setting. Some participants experienced that manually adjusting the flexible wrist was too much effort, although they acknowledged that it was worth the effort for prolonged activities. Several times it was suggested that a myoelectrically controlled wrist might be even more advantageous.

**Table 6:** Reports of advantages, disadvantages and quotes on the wrists (conditions) mentioned in the written responses on the open-ended questions and in the interviews

Wrist (condition)	Advantages	Disadvantages	Quotes	Participant’s preferred choice (n)	Requisites for choosing this wrist
Flex-wrist (static)	- Reliable - Solid while carrying things - No ‘dangling’ - No rist position - Easy to adjust - Handling wheelbarrow is easy	- Difficult to reach narrow spaces - Mechanism switches unwanted - Pressure in socket discourages prosthesis use - Awkward postures → shoulder complaints - During cycling, trembling causes stump-electrode contact → hand opens unintentionally	“I can trust on the knowledge that the wrist is always in the same position.” “I can lean on the hand with full support, without being afraid that the wrist would bend.”	1	N.A.
Flex-wrist (flexible)	- Less restricted in movements - Less unnatural movements - Less burden upper arm and shoulder - Easy to operate - Less effort in prolonged activities - Increased dexterity - Useful in activities: picking up and grabbing tying shoelaces •riding a bicycle •cutting vegetables	- Manually adjusting wrist is too much effort - Increased complaints unaffected side - Change of wrist position occurs unintentionally (i.e., in carrying boxes) → irritating and dangerous - Obliged to think of changing position to neutral after handling - Appearance - Mechanism fragile	“Less awkward movements were required.” “The flexible wrist enabled movements that were not possible before.” “It is too much effort to manually adjust wrist position before handling something. A freely moveable wrist or a wrist than can be adjusted using muscle signals would be better.” “I would have benefitted even more from the flexible wrist if I would still have been employed in a job setting.”	2	- Only flexion positions are enough (1 participant)
Multi-flex wrist (static)	- Sideward moving comfortable (i.e., in leaning on something) and not too compliant - Useful in activities: •riding bicycle •pouring off pans •picking up things •ploughing garden - More natural posture and movements - Less burden shoulder - Less bending of fingers because wrist absorbs forces	- Requires habituation - Missing flexion/extension in driving - Difficult to support paper when writing - Wrists bends when leaning on hands - Sideward movement not comfortable - Missing one’s hold due to unexpected movements	“The continuous presence of the free sideward movements gives me an unsafe feeling. A possibility to fixate this degree of freedom would be of interest.” “Free movement in flexion/extension direction was most important for me.”	0	N.A.
Multi-flex wrist (flexible)	- Moving more naturally, fluently, relaxed; similar as unaffected side - Better posture - Natural appearance - Less burden shoulder - Making activities easier: •Driving •Clean with a vacuum cleaner •Opening door handle •Closing car door •Putting on socks •Close zipper of jacket till top •Support paper when writing •Gardening - Less counter-pressure on stump (i.e., in cycling) - Easy to operate - Wrist does not bend too easy	- Operating buttons is difficult - Moving in flexion/extension and sideward direction is too much; leaning less possible - Freely moveable wrist not favorable in driving and cycling - Mechanism not easy to operate - ‘Fixed’ flexion/extension positions unnatural; operating with unaffected hand - Lack of stability, for example in: •Riding bicycle •Lifting shopping bag	“I got the feeling having to compensate for the movements of the wrist.” “More positions to fixate the wrist in flexion/extension direction would be an improvement.” “Riding a bicycle is really comfortable.” “I realized the major impact of wrist movement; I was grabbing more things and in a more natural way.”	5	- Wrist should have the possibility to fixate flexion/extension and sideward moving independently when necessary (1 participant) - Mechanism should be easier to operate (1 participant)

Note: The advantages, disadvantages and quotes could have been mentioned by more than one participant. The fifth and sixth column indicate how many participants would choose a certain wrist (condition) when getting a new prosthesis, and requisites for their choice

### Compensatory movements

Shoulder joint mobility was normal in all control subjects. All prosthesis users had restricted shoulder exorotation at the affected side. Besides that, one prosthesis user had restricted shoulder function at the unaffected side.

**Fig. 1:**
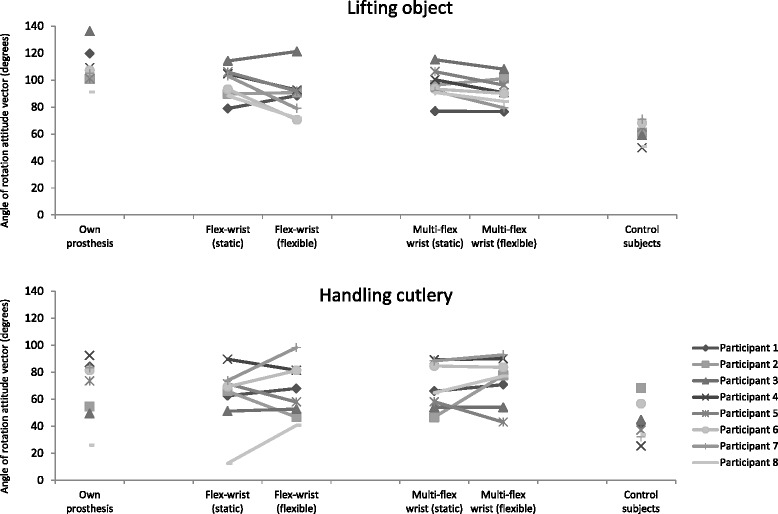
Mean maximum angle of rotation of the shoulder (degrees) for individual participants in different wrist conditions during ADL tasks with small (‘lifting object’, top) and large (‘handling cutlery’, bottom) inter-individual variation

The maximum angles of rotation in the shoulder during execution of the ADL tasks did not consistently differ between static and flexible wrist conditions. Figure 1 shows the maximum angles of rotation in the shoulder joint for all participants in all conditions for two tasks. As shown in Fig. 1, there is a lack of consistent directional response in the shoulder angles from static to flexible wrist conditions over participants.

Table 7 presents the averaged maximum angles of rotation in the shoulder per task for all conditions of the prosthesis users and for the control subjects. It can be clearly seen that the maximum angles for the control participants were smaller in all tasks compared to the prosthesis users, irrespective of wrist condition. The CoV of the maximum angles of rotation varied from 9 to 37 % for the four wrist conditions over the six tasks (Table 7). Lifting an object showed lower CoV (12–18 %), whereas in handling cutlery variability was higher (CoV between 23–37 %). Differences in CoV were also found for prosthesis users using their own prostheses and controls when executing the tasks (CoV was as low as 13 % and as high as 33 % in both groups). These substantial differences between tasks and conditions indicated the lack of consistency in behaviour.

**Table 7:** Maximum angle of rotation of the shoulder (degrees) for all ADL tasks in all wrist conditions of the prosthesis users, and in control subjects

Task	Wrist condition (prosthesis users)					Control subjects
	Own prosthesis	Flex-wrist (static)	Flex-wrist (flexible)	Multi-flex wrist (static)	Multiflex-wrist (flexible)	
Lifting crate	69 ± 11(16 %)	69 ± 10(14 %)	71 ± 7(10 %)	70 ± 12(17 %)	74 ± 13(18 %)	63 ± 8(13 %)
Stirring	73 ± 24(32 %)	89 ± 19(21 %)	81 ± 18(23 %)	94 ± 8(9 %)	85 ± 12(15 %)	46 ± 6(13 %)
Lifting object	109 ± 14(13 %)	97 ± 12(12 %)	88 ± 16(18 %)	96 ± 11(12 %)	91 ± 11(12 %)	61 ± 8(13 %)
Closing zip	64 ± 14(22 %)	61 ± 22(36 %)	52 ± 12(23 %)	53 ± 13(24 %)	65 ± 23(36 %)	32 ± 7(23 %)
Handling cutlery	68 ± 23(33 %)	62 ± 23(37 %)	66 ± 20(30 %)	69 ± 16(24 %)	74 ± 17(23 %)	42 ± 14(33 %)
Turning door handle	49 ± 16(32 %)	51 ± 15(29 %)	53 ± 12(22 %)	52 ± 10(20 %)	47 ± 12(26 %)	34 ± 7(22 %)

Note: Scores are presented as mean ± s.d. (standard deviation) with coefficient of variation in parentheses

## Discussion

This is the first study that compared flexible and static wrist units in an extensive evaluation. Although qualitative data show that prosthesis users tended to be more satisfied with flexible wrists, positive effects of these wrists were hard to objectify, that is, using a wrist with flexion/extension capabilities instead of a static wrist unit did not show substantial differences neither on objective measures of functionality, nor on standardized questionnaires measuring satisfaction, nor on compensatory movements.

Higher satisfaction with flexible compared to static wrist units has been reported previously. From a preliminary field trial in patients using the Multiflex-wrist (Motion Control), it was reported that patients experienced an improvement in overall comfort of the prosthesis and ease of use while performing activities over the course of a day [14]. Similarly, our participants indicated that they were grabbing more objects pro-actively with their prosthesis, that handling objects was easier, and that their dexterity had increased. Another major comment of the prosthesis users was that they experienced a more relaxed and less restricted way of moving. Also, they reported a decrease of awkward shoulder movements and less musculoskeletal complaints. These findings are in line with literature reporting that patients perceived an optimization of their motion pattern when they had more wrist motion capabilities [5], and with the expectation that increased flexibility of allowed wrist motion may avoid awkward shoulder motions [1]. The differences in shoulder angles between prosthesis users and controls were very clear and may be an explanation for the frequently found overuse complaints in prosthesis users. However, in the shoulder movements we did not find consistent differences between static and flexible wrists. Moreover, large variability over participants was observed. One reason for not finding consistent differences in shoulder movements between static and flexible wrists could be that the abundant degrees of freedom in the arm make that compensation is distributed over a series of joint angles and therefore hard to quantify in a specific joint angle. Furthermore, several studies showed that besides shoulder movement, trunk movement is an important compensatory movement in transradial prosthesis users [9–11]. Although we followed the line of previous investigations on the benefits of flexible prosthesis wrists by quantifying shoulder movement, we believe that future studies should also consider trunk motion in their assessment. To extend the investigation of compensatory movements on other outcome measures than joint angles, an objective measure of shoulder and neck muscle activity using electromyography would also be of interest for further research on the benefits of flexible wrists.

Interestingly, although we found positive results in favour of flexible wrists on the qualitative level, these positive effects were not reflected on the quantitative level. We did not find differences for performances on SHAP or B&B. This is contradictory to our expectation that prosthesis users may act faster and easier with their prosthesis using a flexible wrist, and different from earlier findings of Kyberd [4] who reported that an able-bodied participant using a flexible wrist with a prosthesis simulator performed tasks quicker and with less difficulty as a result of wrist flexion, reflected in higher functional scores on the SHAP. The difference between the use of an able-bodied subject and prosthesis users may be part of the explanation of these seemingly contrasting results. Also, participants in the current study used prosthesis hands of different manufacturers. The experience with controlling the experimental hands (which were different from the participants’ own prosthesis hand) and the technical differences in hand opening-closing speed between the used Motion Control and Otto Bock myoelectric hand could have influenced the SHAP and Box and Block scores, since fluency of hand opening and closing is a major factor determining scores on SHAP tasks and on the Box and Block test. Besides that, a previously reported training effect of the SHAP might have played a role in our consecutive testing [25]. These factors, combined with the remarkably large inter-individual variation, could have clouded differences on functionality scores between wrists. Importantly, it could be questioned whether current implementation of clinical measures of functionality with movement speed as primary outcome (i.e., SHAP and Box and Block test) are appropriate to measure changes in functionality resulting from using different wrists. This is emphasized by the discrepancy between the participants’ reports of increased dexterity and ease of use, and the lack of increased scores on the aforementioned objective functionality measures. For future research, an observational measure such as the Assessment of Capacity of Myoelectric Control (ACMC) [26] might be considered when assessing prosthesis functionality with different wrists. However, measures specifically dedicated to evaluate prosthesis wrists should preferably become available in the future.

Measurement instruments were chosen based on the advice of the ULPOM group, the Upper Limb Prosthetic Outcome Measures group [27]. Although this group did not specifically advise evaluation tools for prosthesis wrists, all measures used in the current study have been used extensively in upper limb amputees before. However, the question concerning the appropriateness of measurement instruments could rise again from the lack of significant effects on standardized questionnaires of satisfaction, such as TAPES and D-QUEST, whereas user feedback was very positive about flexible wrists. A closer look at single items of the D-QUEST, however, revealed consistently improved satisfaction scores in favour of flexible wrists, such as ‘ease of adjusting’. Many items of D-QUEST and TAPES concern the prosthesis (hand) in general and as a whole. As the wrist is a relatively ‘small’ component of the prosthesis, the general character of questionnaires like D-QUEST and TAPES may explain why improvements in satisfaction as a result of the wrist are not reflected in end scores on these questionnaires. Again, new versions of tests evaluating prosthetic components might consider including items related to wrist performance.

A limitation of the study might have been the short periods of trying out the different wrist conditions. It could be questioned whether periods of two weeks were long enough to observe changes in functionality and satisfaction when testing new devices like prosthesis wrists. Two weeks is definitely too short to objectively measure long-term changes such as a decrease in musculoskeletal complaints. Also, a lack of a training in which the users get specific directions on how to implement the advantages of the flexible wrists and how to reduce compensatory movements, might be an explanation for similar results. Especially experienced prosthesis users who have reached a steady state performance with their own prosthesis might not be able to change their long-standing movement patterns in such a short period. In the interviews, participants indicated they had enough time to get used to the device and used it under different circumstances. As such, we are confident that the prosthesis users in our study were able to at least indicate advantages, disadvantages and ease of use after exploring the possibilities of the new device during two weeks. A further limitation of the study might be the relatively small number of participants. The inter-individual variation turned out to be quite high in some of the outcome measures, which may have been one of the causes for not finding effects of flexible wrists on objective measures. Our choice for using ADL tasks may also be a source of the high inter-individual variation on the outcome measures of compensatory movements, although we standardized the tasks as much as possible.

A strength of the study was the application of a combination of quantitative and qualitative measurements instruments. Where the quantitative measures did not show differences over the four wrist conditions, user feedback mostly underlined the benefits of a relatively small extra investment like a flexible wrist. The named advantages and disadvantages in the open-ended questions and interviews may be valuable information for manufacturers and especially clinicians, when advising the purchase of a prosthesis wrist. Before a definite choice for a wrist can be made, the personal preference for wrist flexibility or stability during daily life activities or leisure time activities should become clear. A try-out period with both types of wrists could be useful to determine such a preference. Since our participants indicated that type of work is also an important determinant for experiencing benefit from either a flexible or static wrist, such a try-out period could also provide information on the necessity for stability or flexibility during work related activities.

## Conclusions

Functionality with flexible or static wrist units, assessed with standardized outcome measures, did not differ in transradial amputees using a myoelectric prosthesis. However, users tended to be more satisfied with the ease of use and way of moving when they used a flexible compared to a static prosthesis wrist. Our results emphasize the importance of developing and choosing suitable clinical measures to evaluate new prosthetic devices. Although most prosthesis users seem to benefit from flexible wrists more than from static wrists, it is important to take into account a person’s needs, situation, work and skills when prescribing a prosthesis wrist.

## Appendix

**Table 8:** Description of ADL tasks used to assess shoulder movements

ADL	Task description
Turning door handle - one-handed - standing	A door-handle, mounted on a wooden post at 24 cm high, was fixed at the corner of a foldable crate. Participants were standing with their heels at a taped line at 35 cm from the edge of the table. The door handle was placed right in front of the participants’ affected arm at 25 cm from the edge of the table. Participants turned the door handle down and up with their prosthesis hand.
Lifting crate - two-handed - standing	Participants were standing with their heels at a taped line at 35 cm from the edge of the table. They lifted a foldable crate of 37 x 25 x 19 cm (length x width x height, total weight 1,54 kg) with both hands from the affected to the unaffected side, over a box of 29 x 29 x 17 cm (length x width x height) that was located in front of the participant, at the edge of the table. Tape signs at 20 cm left and right of the box indicated start and end position of the crate. The crate had to be placed beyond these signs.
Closing and opening zip - two-handed - standing	Participants were fitted with a custom-made jacket with a zip (22 cm long) at the front, starting at hip height. Participants grabbed the zipper with their prosthesis hand and closed and opened the zip, while supporting the jacket with their unaffected hand. Participants were allowed to pass the zipper with their unaffected hand to their prosthesis hand when grabbing it.
Lifting object - one-handed - seated	A box of 29 x 29 x 17 cm (length x width x height) was placed in front of the participant, at the edge of the table. A wooden cylinder (7 cm diameter, 14 cm high) was placed 14 cm next to the wooden box and 14 cm from the edge of the table, at the affected side of the participant. The participant grabbed the cylinder at its upper side and placed it on the top of the box, at the marked middle point.
Stirring into cup - one-handed - seated	A cup filled with water was placed in front of the participant at 25 cm from the edge of the table. Participants picked up the spoon with their prosthesis hand, stirred 5 times, and released the spoon. Participants were allowed to pass the spoon with their unaffected hand to their prosthesis hand when grabbing it.
Handling cutlery - two-handed - seated	A piece of clay (5 cm long) was placed in front of the participant at 25 cm from the edge of the table. Participants held a fork in their prosthesis hand and a knife in their unaffected hand. From this starting position, participants fixated the clay with the fork, and cut it with the knife.
